# Improving the resident assessment process: application of App-based e-training platform and lean thinking

**DOI:** 10.1186/s12909-023-04118-2

**Published:** 2023-02-28

**Authors:** Wenqing Yuan, Zhengqian Li, Jiangli Han, Hongling Chu, Shan Lu, Shixian Gu, Ning Shen

**Affiliations:** 1 Department of Education, Peking University Third Hospital, 100191 Beijing, China; 2 Department of Anesthesiology, Peking University Third Hospital, Beijing, 100191 China; 3 Department of Cardiology, Peking University Third Hospital, Beijing, 100191 China; 4 Research Center of Clinical Epidemiology, Peking University Third Hospital, Beijing, 100191 China; 5 Department of Pulmonary and Critical Care Medicine, Peking University Third Hospital, 100191 Beijing, China

**Keywords:** Standardized resident training, Online assessment, Lean thinking, Process improvement

## Abstract

**Background:**

The assessment system for standardized resident training is crucial for developing competent doctors. However, it is complex, making it difficult to manage. The COVID-19 pandemic has also aggravated the difficulty of assessment. We, therefore, integrated lean thinking with App-based e-training platform to improve the assessment process through Define–Measure–Analyze–Improve–Control (DMAIC) cycles. This was designed to avoid unnecessary activities that generate waste.

**Methods:**

Panels and online surveys were conducted in 2021–2022 to find the main issues that affect resident assessment and the root causes under the frame of waste. An online app was developed. Activities within the process were improved by brainstorming. Online surveys were used to improve the issues, satisfaction, and time spent on assessment using the app.

**Results:**

A total of 290 clinical educators in 36 departments responded to the survey, and 153 clinical educators used the online app for assessment. Unplanned delay or cancellation was defined as the main issue. Eleven leading causes accounted for 87.5% of the issues. These were examiner time conflict, student time conflict, insufficient examiners, supervisor time conflict, grade statistics, insufficient exam assistants, reporting results, material archiving, unfamiliarity with the process, uncooperative patients, and feedback. The median rate of unplanned delay or cancellation was lower with use of the app (5% vs 0%, *P* < 0.001), and satisfaction increased (*P* < 0.001). The median time saved by the app across the whole assessment process was 60 (interquartile range 60–120) minutes.

**Conclusions:**

Lean thinking integrated with an App-based e-training platform could optimize the process of resident assessment. This could reduce waste and promote teaching and learning in medical education.

## Introduction

All medical students have to participate in resident training to become competent doctors. In China, medical education is a multi-track, long-term process that starts at the undergraduate level, and includes further stages at the graduate, doctoral, and post-doctoral levels. In 2013, to ensure the quality and homogeneity of medical staff, and improve the national quality of the health service as a whole, seven Chinese government ministries jointly launched the Guidelines for Standardized Resident Training (SRT) [1]. These guidelines require all clinicians with a bachelor’s degree or above to receive standardized training for residents. Based on the concept of synergy between clinical practice and college education, Peking University Health Science Center developed a training model for clinical medicine students known as the four-in-one track, which has been in place since 1997. It was the first in China to combine standardized training for four types of students: clinical medicine graduate students, eight-year undergraduate medical students, students with equivalent educational qualifications and residents. All four types of students need to receive the same level of training and reach the same level of competence in medicine. To develop competent [2] physicians in clinical medicine, standardized resident training in China has paid increasing attention to the process of assessment. This has promoted residents’ capacity to apply their knowledge and skills to actual clinical practice.

The process of standardized resident training is very complex. It involves students rotating through dozens of clinical departments. It also involves a wide range of people, such as clinical educators, hospital administrators, and patients. The overall process takes a lot of time and energy for its design, proposition, organization, evaluation, feedback, and filing [3]. New SRT assessment tools were proposed to simplify the process and to improve the quality [4–6]. During the COVID-19 pandemic, implementing the assessment process became more difficult because of the increased pressure on both clinical educators and students. For standardized resident training, assessment is critical [7]. However, the integrity and standardization of actual practices are often compromised because of the complexity of the process, clinical work stress, and the time required.

Lean thinking is a management framework originated from a car manufacturing business that aims to help practitioners improve efficiency and the quality of work. Womack first proposed lean thinking as a management concept and methodology in 1991 [8]. Lean thinking is the strategic dimension of the concept of lean [9] and focuses on optimizing processes, improving efficiency, and providing continuous improvements for all members of the organization as a whole by removing waste [10]. On the basis of the concept of waste put forward by Ohno [11], Shingo [12] identified the seven classical waste types from manufacturing, which are overproduction, inventory, over-processing, motion, defects, waiting and transportation. In particular, under-used talent has been further identified as an eighth waste [13]. In addition to its beneficial application in manufacturing, lean thinking has been widely used and validated useful in a wide variety of fields including educational settings [14, 15]. In higher education, more work has been carried out to define sub-wastes and explain the eight wastes [16]. Recent years have seen an emerging use of lean thinking in medical education [17–20]. The philosophy and methodology to identifying and eliminating waste generated by massive interdepartmental interaction activities and processes could allow universities and teaching hospitals to manage the SRT assessment in an optimized way.

This study focused on streamlining the process of resident assessment by using an App-based e-training platform, drawing on the philosophy of lean thinking. This study is important because it explores how to integrate lean thinking with informational technologies and sheds light on the value of adopting lean thinking for resident assessment. This has significant practical implications in the field of medical education under the impact of COVID-19. It therefore enriches the literature by providing a more efficient management framework for resident assessment for use in teaching hospitals.

## Methods

### Optimized SRT assessment process establishment through DMAIC cycle

**Fig. 1:**
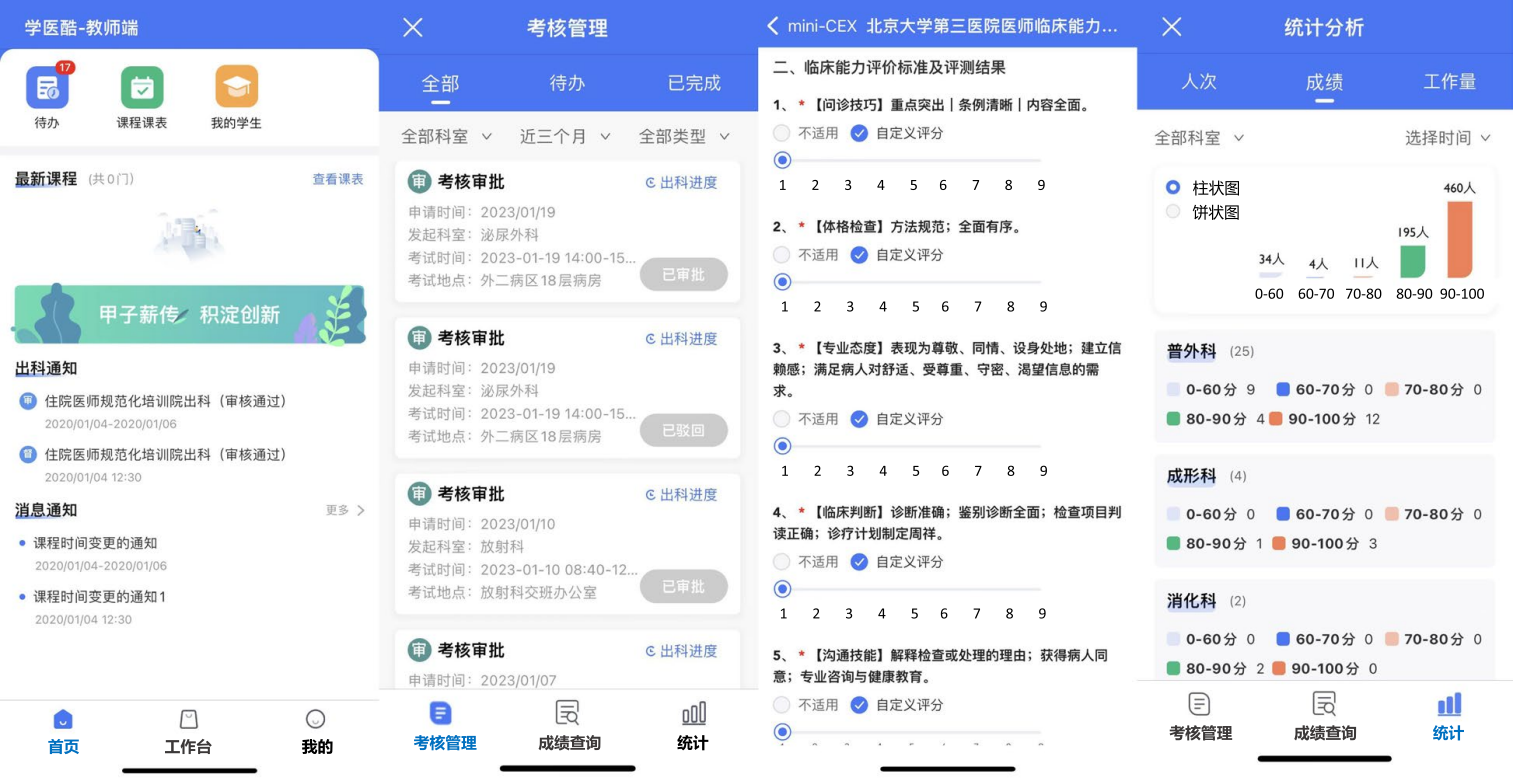
Chinese User interface of “XUEYIKU” presented on a mobile device

This study followed the lean six sigma DMAIC methodology [21] to optimize the process of resident assessment using an App-based e-training platform, a mobile app called “XUEYIKU” developed by Peking University Third Hospital (Fig. 1). DMAIC refers to the five sequential phases of process improvement: Define, Measure, Analyze, Improve, and Control [22]. The DMAIC tool of SigmaGuide (http://www.sigmalogic.de) was used to help implement the DMAIC cycle. It supports collecting, processing and integrating the necessary information along the five phases of DMAIC and provide tools and charts along the DMAIC phases.

#### Define

**Fig. 2:**
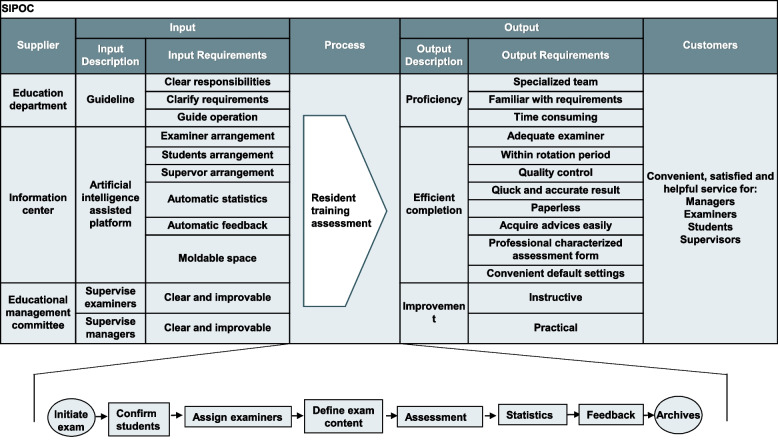
SIPOC diagram of resident assessment (the process shown is the pre-study process)

Panels hearing from students and teachers about resident assessments were held every month. Complaints and opinions were filtered and translated into requirements. It was confirmed that the key need shared by both clinical students and educators was to have no unplanned delay or cancellation (UDC) of resident assessment. To integrate and clarify the scope of this study, the core process was mapped to the Supplier–Input–Process–Output–Customer (SIPOC) diagram (Fig. 2). A study team of multidisciplinary experts including administrators, clinical teachers, and evaluation experts was established. The main issue or defect was defined as UDC, which results in unnecessary or additional work. The definition of the rate of UDC is cases of UDC/total planned resident assessments per semester. After comprehensive consideration based on the baseline data, the target of this study was set as reducing the incidence of UDC to zero by March 2022.

#### Measure

In this project, the pre-study process started with an exam and ended with the filing of examination materials (Fig. 2). Factors causing delay or cancellation were analyzed across the process using the wastes framework (Table 1). In total, 18 sub-wastes were identified through brainstorming of Input–Analyze, with actual examples after 26 field observations and 16 team meetings since June 2021. Online surveys developed by the study team were conducted using random sampling in 36 clinical departments to identify the negative factors influencing resident assessment. We then compared the rate of UDC, satisfaction with the whole process of resident assessment (using a five-point Likert-type scale), and time savings before and after the application of an online app developed using informational technology (See Improve).

**Table 1:** Wastes occurring in resident assessment

Types	Sub-wastes	Examples from teaching hospitals
Waiting	Prepare test papers	Choose and generate test questions Test paper and assessment form formation Printing and bookbinding of test papers and assessment form
	Responses	Waiting for responses from examiners Waiting for responses from students Waiting for responses from supervisors Waiting for responses from patients
	Approvals	Hierarchy review Manual signature
	Conflicts	Examiner change due to time conflict Classroom change due to arrangement conflict
	Grades and scores	Mark test papers Record the score of a batch of test papers manually Statistical analysis of the result Report and feedback
Over-processing	Multiple handovers	Too many people or manual work involved in process steps
	Repeated notification	Same information being manually notified to all kinds of personnel (examiners, students, supervisors, and administrators) Print the test paper repeatedly
	Unnecessarily repeated tasks	Print the assessment form repeatedly
Overproduction	Produce extra useless information	Generate unnecessary information about students and examiners
	Excessive participation	Too much human power with the same goals
Inventory	Save extra materials	Unnecessarily save copies of the same test papers or assessment forms
	Excessive storage time	Maintaining space for test-related paper materials
Transportation	Devices stored away	Printer is at another place Computers for statistical analysis is in another place
Motion	Excess movements	Students return from another rotated department due to delay in examination Signed documents moving from site to site
Defects	Understanding deviations	Misunderstandings of regulations and requirements Wrong translation of examiners’ or supervisors' opinion Unrecognized written words from examiners and supervisors
	Errors in the data processing	Wrong grades in the system Wrong statistical methods
Under-utilized talent	Consume enthusiasm	Redundant process leads to waste of time and emotional exhaustion
	Information & technology	Information talents were not involved Technology talents were not involved

#### Analyze

**Fig. 3:**
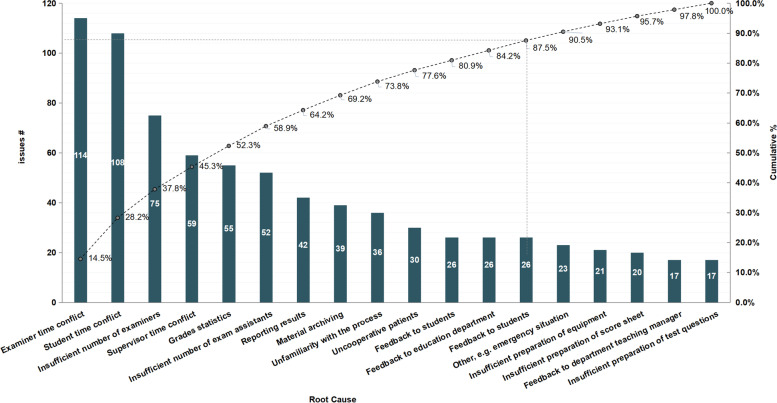
Root causes of unplanned delay or cancellation (UDC) of resident assessment in a teaching hospital. The vertical left axis represents the frequency of occurrence of root causes of UDC of resident assessment which are listed in descending order of counts starting at the left side of the Pareto chart. The right vertical axis represents the total cumulative percentage of root causes of UDC. The crossed dotted lines indicate that eleven leading causes accounted for 87.5% of the issues

The collected data on factors causing UDC were evaluated graphically. Eleven main root causes were responsible for 87.5% of the issues encountered. These were examiner time conflict, student time conflict, insufficient number of examiners, supervisor time conflict, grade statistics, insufficient number of exam assistants, reporting of results, material archiving, unfamiliarity with the process, uncooperative patients, and feedback (Fig. 3).

#### Improve

**Fig. 4:**
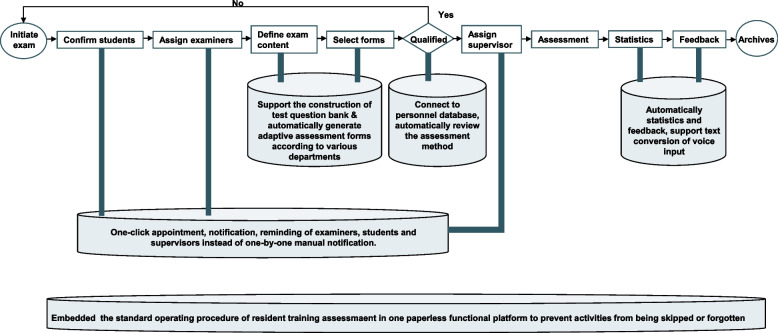
The improved process for resident assessment, showing the value of IT. The whole process of resident assessment has been improved under the philosophy of lean. Each activity supported by the App-based e-training platform is connected to a can with detailed description

To find solutions for the root causes, we reviewed and analyzed the process map in a brainstorming session, to find intelligence technology (IT)-supported ways to improve activities (Fig. 4). We established an App-based e-training platform using the lean thinking in order to improve resident training assessment. To be specific, we used natural language processing (NLP) technology [23–26] to extract features and convert them to a normalized data structure. The department of education provided training on how to organize the resident assessment.

As we previously reported [27], NLP algorithm in the current study is mainly used after assessment and serves the feedback stage (Fig. 4). Taking a resident's inadequate grasp of the differential diagnosis of systemic lupus erythematosus in the exam as an example, the XUEYIKU app divided the text of a teacher's evaluation into words in a Chinese word segmentation process, and extracted the keywords "systemic lupus erythematosus” and “differential diagnosis" by using the semantic analysis of NLP. Specifically, abbreviation substitution, synonym replacement, and text similarity calculation may also be used in information retrieval, machine translation, text mining etc., according to the teacher's actual evaluation. The student side of the app matches training tasks with the same theme in the test question bank according to the keywords extracted and sends them to students for intensive learning.

The measured main root causes related to multiple aspects in the wastes framework (Table 1). Time conflicts of examiners, students, and supervisors together accounted for 40.8% (281/688) of the main causes. These causes led to waiting, over-processing and motion. The new App-based e-training platform supports one-click notification, educator database memory and automatic student rotation schedule-matching. Functions to support grade statistics, reporting and feedback were developed to save waiting, and have solved 25.4% (175/688) of the main causes. Text conversion of voice input has also been developed to save time and provide more feedback.

Insufficient examiners and exam assistants together accounted for 18.5% (127/688) of the main causes. These causes showed under-used talent in resident assessment. The IT-supported online platform included both information and technology talents, and it has optimized the whole process to assist the organization of assessment.

Archiving accounted for 5.7% (39/688) of the main causes. The app was designed to file all trace and assessment forms, grades and feedback. It has therefore helped to solve the inventory problem of material archiving. Standard operation procedure (SOP) and communication skills were both highlighted in the training to help reduce the problems of process unfamiliarity (5.2%, 36/688) and uncooperative patients (4.4%, 30/688). Step-by-step activities are also embedded in the app to aid the completion of the whole process.

#### Control

The study team verified the improvements and benefits of the newly developed app continuously. They also organized feedback panels and discussions over an interval of 2–4 weeks. An SOP was developed, including a process control, and this was installed and revised to take into account field observations. Control charts were used to monitor the process, and departments with high rates of UDC were prompted about SOP use by the educational management committee.

### Evaluation of the process established

An online survey was conducted to investigate the effect of introducing the optimized SRT assessment process. We compared the rate of UDC and the satisfaction before and after the use of the App-based e-training platform using the Wilcoxon matched-pairs signed-ranks test. All data was analyzed using Stata version 15.0. All tests were two-sided and were considered statistically significant if *P* < 0.05.

### Ethical review

This study was reviewed and approved by the Peking University Third Hospital Medical Science Research Ethics Committee (No. IRB00006761-M2022063). All methods were carried out in accordance with relevant guidelines and regulations. Informed consent was obtained from all participants.

## Results

The SOP for resident training assessment was built using a self-developed mobile app called “XUEYIKU”. Quality control measures were divided into three areas: training for organizers and examiners, field supervision by the educational management committee and real-time feedback data obtained through the examination system. The XUEYIKU app was officially used in June 2020. Until 2022, there was a total of 6392 medical students and 6616 teachers from 29 different departments registered online.

A total of 290 clinical educators in 36 departments responded to the online survey. Before using the online mobile app, all participants gave causes for UDC. In total, 153 participants used the app for resident assessments. The median rate of UDC before and after the introduction of the app was 5% (0%, 15%) and 0% (0%, 10%) (*P* < 0.001) (Table 2). There was a statistically significant difference in satisfaction before and after the introduction of the app (*P* < 0.001) (Table 3). The median time saved by the app was 60 (interquartile range 60–120) minutes. The study team observed more friendly and positive communications between clinical educators and students after the improvement of the resident assessment process.

**Table 2:** Comparison of rate of UDC before and after app introduction in various departments (*N* = 153)

Department/Disciplines	Before (%)	After (%)	P
Internal medicine	0 (0, 10)	0 (0, 10)	0.001
Surgery	10 (0, 20)	0 (0, 10)	< 0.001
Obstetrics and Gynecology	0 (0, 10)	0 (0, 5)	0.046
Pediatrics	0 (0, 15)	0 (0, 10)	0.159
Imaging, laboratory and pathology	0 (0, 20)	0 (0, 10)	0.0049
Others (Ophthalmology, Infectious diseases, Oncology and etc.)	10 (0, 10)	0 (0, 10)	0.001
Total	5 (0,15)	0 (0, 10)	< 0.001

**Table 3:** Comparison of satisfaction before and after app introduction in various departments (*N* = 153)

Department/Disciplines	After-Before	P
Internal medicine	0 (0, 0)	0.666
Surgery	0 (0, 1)	0.014
Obstetrics and Gynecology	0 (0, 0)	0.317
Pediatrics	0 (0, 0)	0.317
Imaging, laboratory and pathology	0 (0, 1)	0.058
Others (Ophthalmology, Infectious diseases, Oncology, and etc.)	0 (0, 1)	0.063
Total	0 (0, 1)	0.0027

## Discussion

### Optimizing the process of resident assessment to provide continuous improvements in quality

COVID-19 has greatly influenced the field of education and has highlighted a profound need for advanced technology. In teaching hospitals, educators are also clinical doctors. In medical education, face-to-face assessment is still the dominant way for clinical educators to evaluate whether residents are competent. In this study, the pre-study process for resident assessment was optimized and improved by eliminating root causes leading to UDC, all of which could cause wasting of time and resources, and loss of enthusiasm. A total of 149 resident assessments were held from June 2021 to January 2022. A 5% UDC means five to six cancellations of examinations per semester. One cancellation involves at least three groups of people, including clinical doctors, residents or students and supervisors from the educational management committee. It generates schedule confusion, requires rearrangement of the examination site and may result in emotional exhaustion for the organizer, who may be under considerable pressure from both clinical and educational work. It is therefore necessary to optimize the complex process of resident assessment to provide continuous quality improvement and support the development of medical talent. The first step in optimizing any process is to eliminate or reduce the root causes of the main problem. We found 11 root causes, which could be classified into waiting, over-processing, inventory, motion, defects, and under-used talent using the framework of waste from lean philosophy. The application of an App-based e-training platform, together with supportive training and the development of an SOP helped to reduce and eliminate these root causes. UDCs have been significantly reduced, especially in departments requiring surgical operations. These departments often have less time to spend on clinical education. Some activities like feedback were being compressed because of limited time. The app helped to optimize the whole process and enable clinical doctors to complete the assessment process.

### Integration of lean thinking and App-based e-training platform to improve efficacy

Lean thinking is both a methodology and a philosophy, designed to build continuous quality improvement in the culture of an organization. By eliminating factors hindering the overall process of resident assessment, the combination of lean thinking and App-based e-training platform in this study helped to build a more user-friendly and efficient process. Our work confirms the efficacy of integrating lean thinking and App-based e-training platform, and sheds light on how to improve a complex assessment process by preventing waste.

Using the waste framework, we were able to save time through multiple activities supported by the app. For instance, the function of sending notifications to everyone involved in the examination saved time spent on phone calls or text messages. The function providing grade statistics and reports saved doctors from calculating or preparing those manually, avoiding potential errors. The function of text conversion from voice input could save time and enable more detailed feedback. The total time saved was a median of about 60 min, which was equal to the time a doctor spends on two laparoscopic appendectomy surgeries or four hysteroscopic surgeries. The time saved could also be spent in improving teaching or assessment quality. Time cost reduction, process optimization, and satisfaction improvements could all contribute to improved efficacy of the assessment process and result in more competent doctors.

### Fostering a teaching and learning culture

The app helped clinical educators to avoid missing activities in the assessment process, because the steps have to be completed sequentially. This therefore reduces the requirement to remember steps and also avoids any confusion. The informational staff at the hospital have also been encouraged to contribute to medical education, and a culture of full participation of clinical educators, administrators, and technicians in teaching has been gradually developed. This suggests that the introduction of lean thinking can increase satisfaction and enthusiasm in the management of medical education. It supports the organization’s approach to quality, and once the culture has been built, a mutually beneficial reinforcing cycle of lean and quality improvement for both educators and students should be developed.

### Limitations

To the best of our knowledge, this is the first prospective study to integrate lean thinking with App-based e-training platform to improve the process of resident assessment. However, this study had some limitations. First, it was based on a single institution. The hospital is representative of most teaching hospitals in China, because clinical medicine at Peking University plays a leading role in the field of medical education. Nevertheless, the extrapolation of the study findings might be limited by other factors including financial investment in education, informational level and the culture of teaching in other hospitals. Second, there might be recall bias, because the UDC rate was provided by clinical educators. An error log is needed to record objective data on routine administration. Additionally, the COVID-19 pandemic might have compounded the negative impact of examination cancellations and influenced the causes leading to UDC.

## Conclusions

Combination use of DMAIC cycle framework and App-based e-training platform has a significant positive effect in optimizing the process of resident assessment. This integration reflects the successful use of lean thinking in medical education, and encourages a stronger teaching environment and culture for both students and clinical educators.

## Data Availability

All data generated or analysed during this study are included in this published article. A license was required to use any of the data collection instruments in this study upon reasonable request and with permission of the authors (contact email: yuanwenqing@bjmu.edu.cn).
